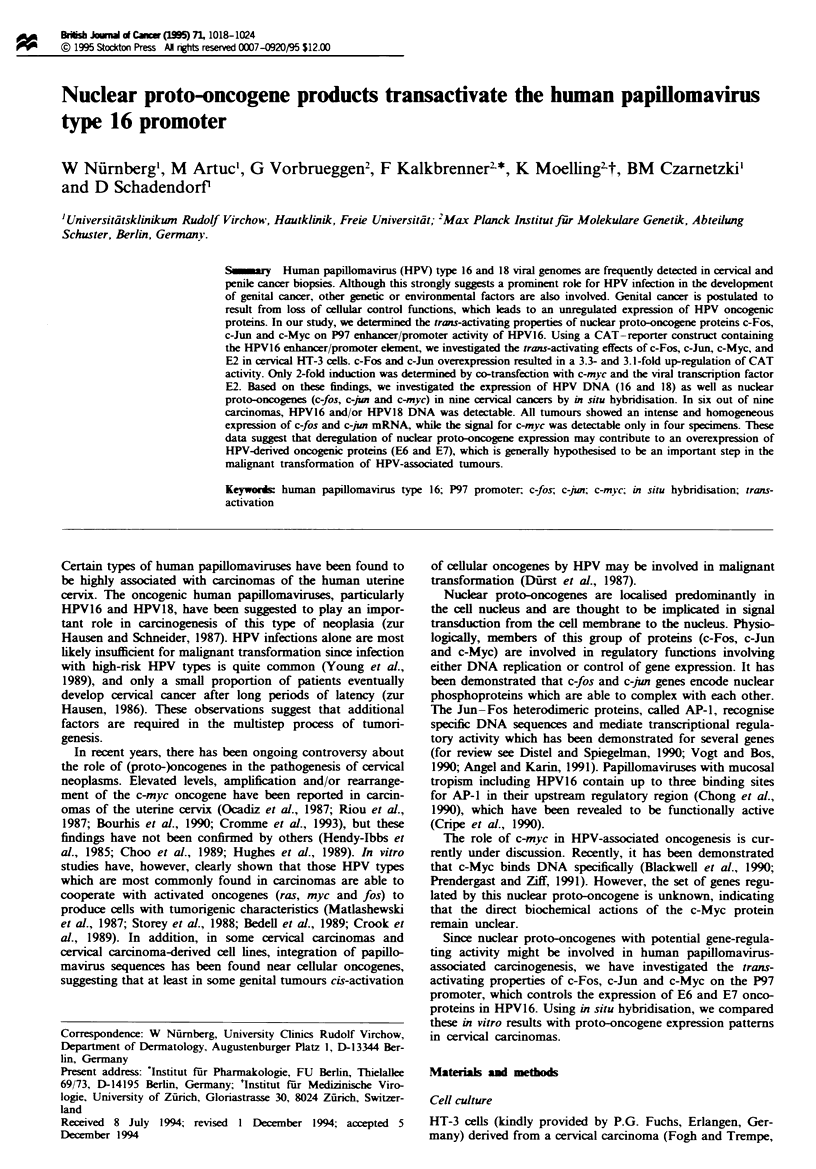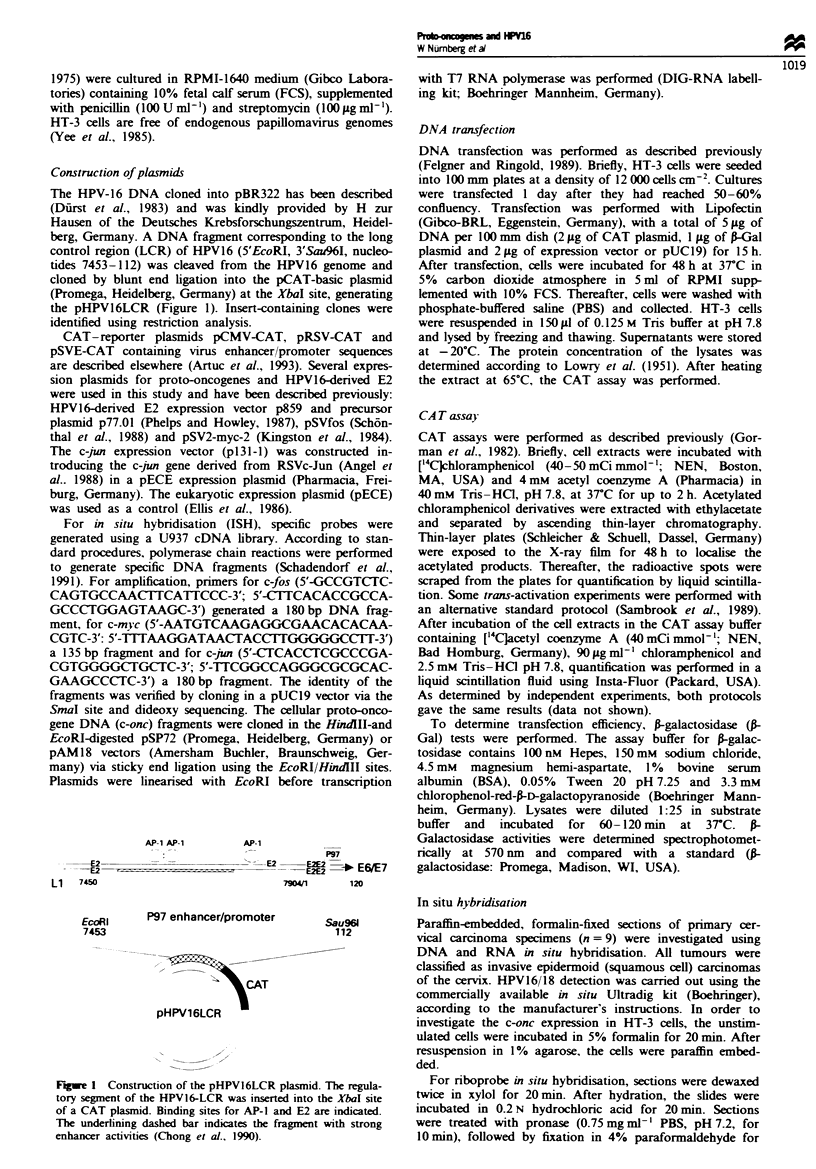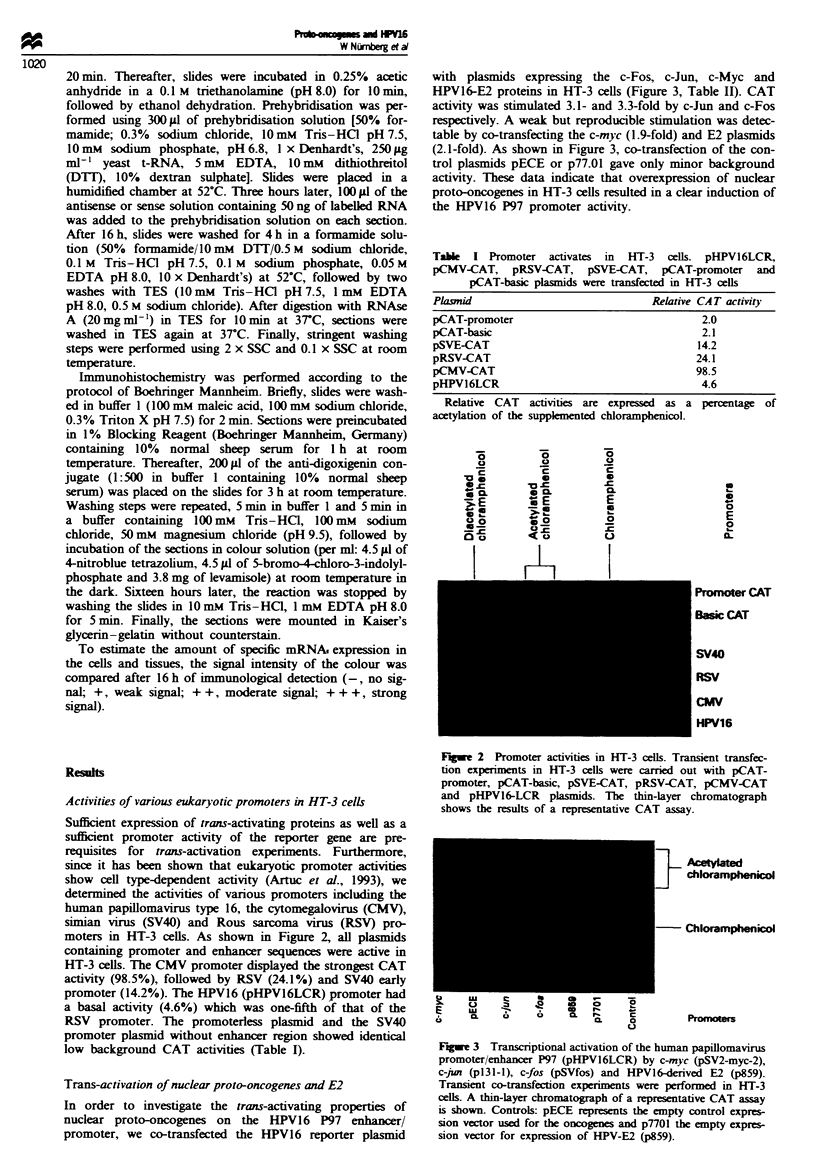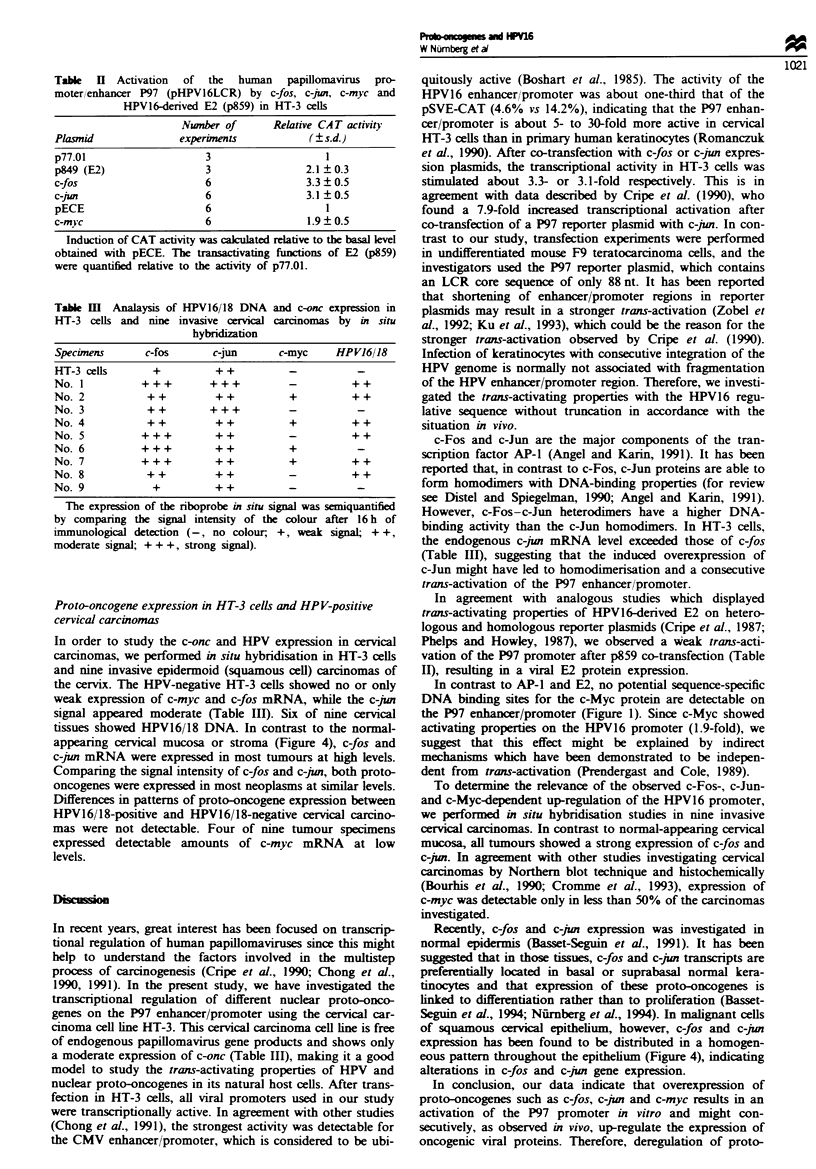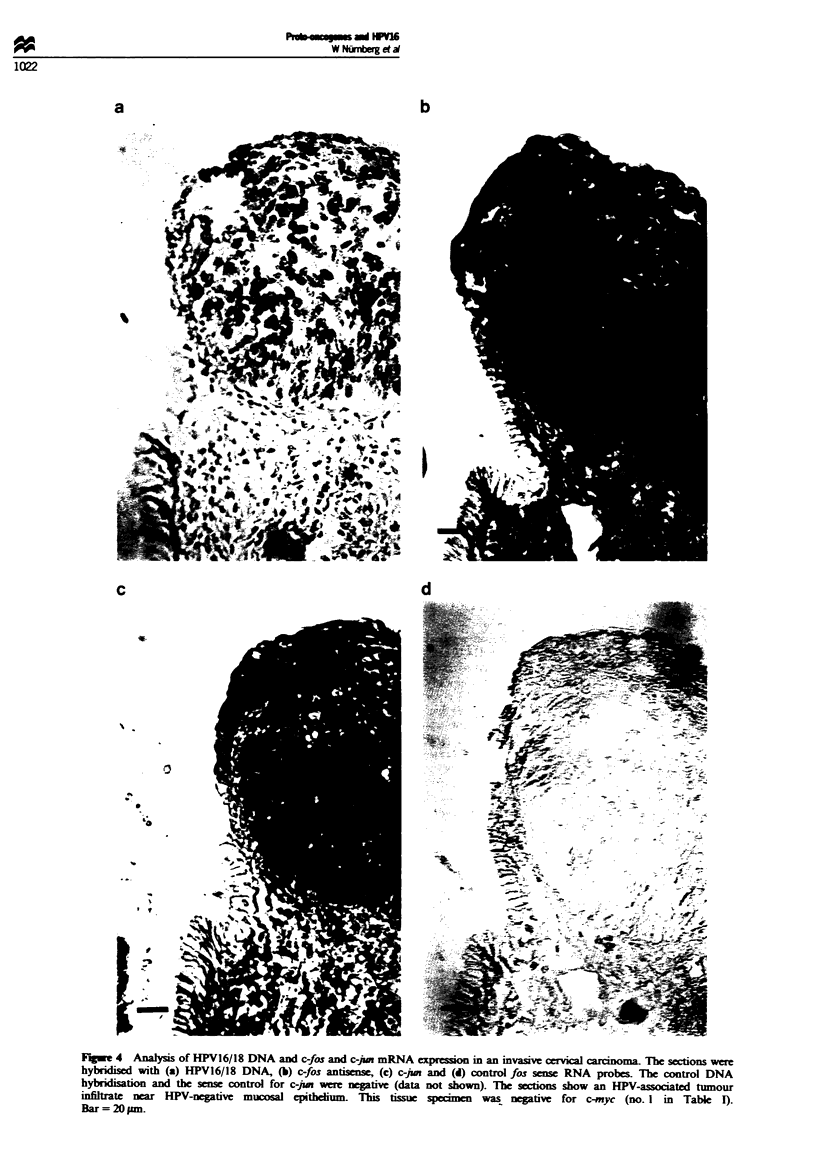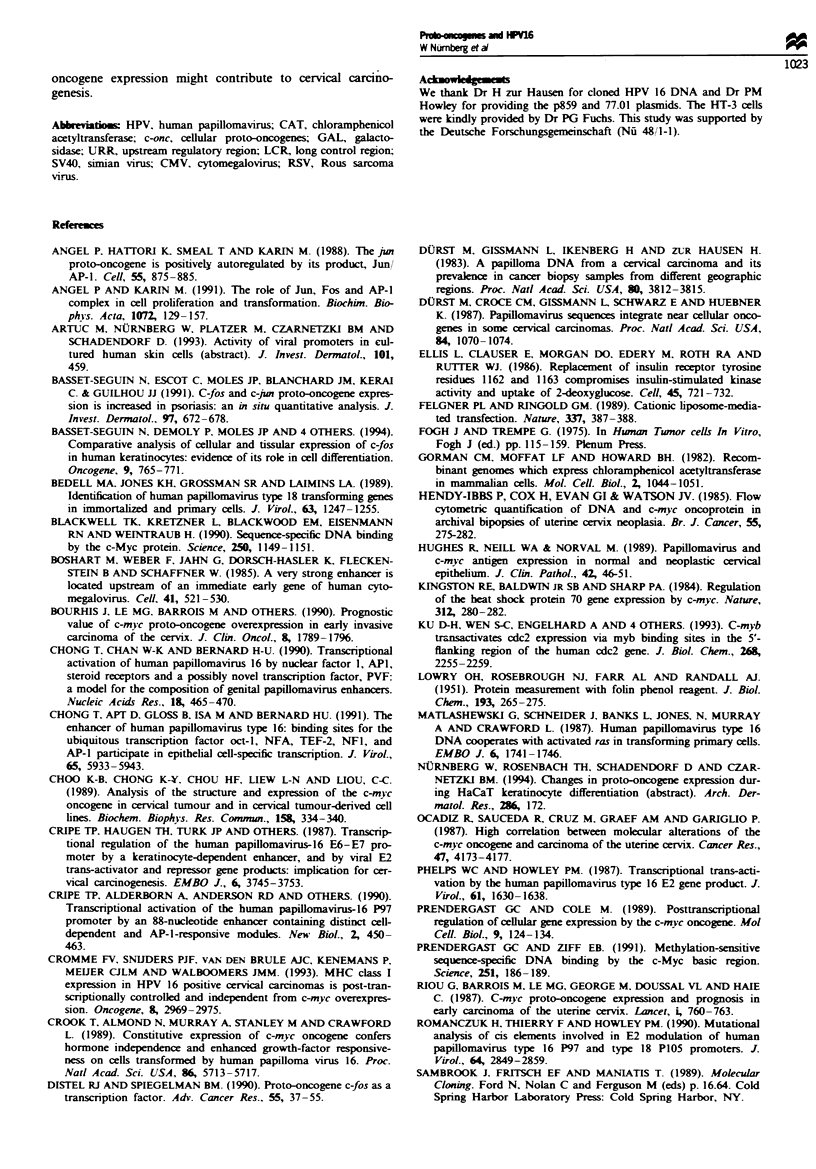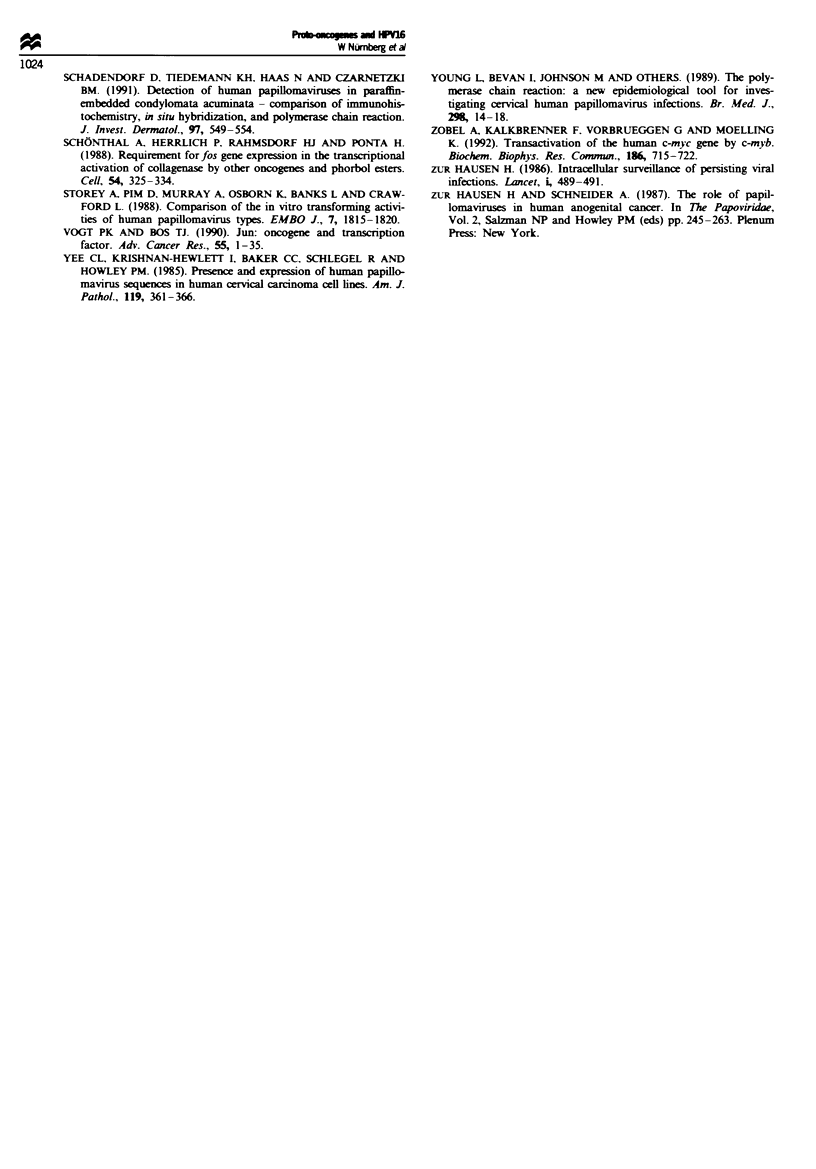# Nuclear proto-oncogene products transactivate the human papillomavirus type 16 promoter.

**DOI:** 10.1038/bjc.1995.196

**Published:** 1995-05

**Authors:** W. Nürnberg, M. Artuc, G. Vorbrueggen, F. Kalkbrenner, K. Moelling, B. M. Czarnetzki, D. Schadendorf

**Affiliations:** Universitätsklinikum Rudolf Virchow, Hautklinik, Freie Universität, Berlin, Germany.

## Abstract

**Images:**


© 1995 Stockton Press All rights reserved 0007-0920/95 $12.00

### Nuclear proto-oncogene products transactivate the human papillomavirus type 16 promoter

W Nürnberg[1], M Artuc[1], G Vorbrueggen[2], F Kalkbrenner[2,*], K Moelling[2,†], BM Czarnetzki[1] and D Schadendorf[1]

*[1]Universitätsklinikum Rudolf Virchow, Hautklinik, Freie Universität; [2]Max Planck Institut für Molekulare Genetik, Abteilung Schuster, Berlin, Germany.*

**Summary** Human papillomavirus (HPV) type 16 and 18 viral genomes are frequently detected in cervical and penile cancer biopsies. Although this strongly suggests a prominent role for HPV infection in the development of genital cancer, other genetic or environmental factors are also involved. Genital cancer is postulated to result from loss of cellular control functions, which leads to an unregulated expression of HPV oncogenic proteins. In our study, we determined the *trans*-activating properties of nuclear proto-oncogene proteins c-Fos, c-Jun and c-Myc on P97 enhancer/promoter activity of HPV16. Using a CAT-reporter construct containing the HPV16 enhancer/promoter element, we investigated the *trans*-activating effects of c-Fos, c-Jun, c-Myc, and E2 in cervical HT-3 cells. c-Fos and c-Jun overexpression resulted in a 3.3- and 3.1-fold up-regulation of CAT activity. Only 2-fold induction was determined by co-transfection with c-*myc* and the viral transcription factor E2. Based on these findings, we investigated the expression of HPV DNA (16 and 18) as well as nuclear proto-oncogenes (c-*fos*, c-*jun* and c-*myc*) in nine cervical cancers by *in situ* hybridisation. In six out of nine carcinomas, HPV16 and/or HPV18 DNA was detectable. All tumours showed an intense and homogeneous expression of c-*fos* and c-*jun* mRNA, while the signal for c-*myc* was detectable only in four specimens. These data suggest that deregulation of nuclear proto-oncogene expression may contribute to an overexpression of HPV-derived oncogenic proteins (E6 and E7), which is generally hypothesised to be an important step in the malignant transformation of HPV-associated tumours.

**Keywords:** human papillomavirus type 16; P97 promoter; c-*fos*; c-*jun*; c-*myc*; *in situ* hybridisation; *trans*-activation

Certain types of human papillomaviruses have been found to be highly associated with carcinomas of the human uterine cervix. The oncogenic human papillomaviruses, particularly HPV16 and HPV18, have been suggested to play an important role in carcinogenesis of this type of neoplasia (zur Hausen and Schneider, 1987). HPV infections alone are most likely insufficient for malignant transformation since infection with high-risk HPV types is quite common (Young *et al.*, 1989), and only a small proportion of patients eventually develop cervical cancer after long periods of latency (zur Hausen, 1986). These observations suggest that additional factors are required in the multistep process of tumorigenesis.

In recent years, there has been ongoing controversy about the role of (proto-)oncogenes in the pathogenesis of cervical neoplasms. Elevated levels, amplification and/or rearrangement of the c-*myc* oncogene have been reported in carcinomas of the uterine cervix (Ocadiz *et al.*, 1987; Riou *et al.*, 1987; Bourhis *et al.*, 1990; Cromme *et al.*, 1993), but these findings have not been confirmed by others (Hendy-Ibbs *et al.*, 1985; Choo *et al.*, 1989; Hughes *et al.*, 1989). *In vitro* studies have, however, clearly shown that those HPV types which are most commonly found in carcinomas are able to cooperate with activated oncogenes (*ras*, *myc* and *fos*) to produce cells with tumorigenic characteristics (Matlashewski *et al.*, 1987; Storey *et al.*, 1988; Bedell *et al.*, 1989; Crook *et al.*, 1989). In addition, in some cervical carcinomas and cervical carcinoma-derived cell lines, integration of papillomavirus sequences has been found near cellular oncogenes, suggesting that at least in some genital tumours *cis*-activation of cellular oncogenes by HPV may be involved in malignant transformation (Dürst *et al.*, 1987).

Nuclear proto-oncogenes are localised predominantly in the cell nucleus and are thought to be implicated in signal transduction from the cell membrane to the nucleus. Physiologically, members of this group of proteins (c-Fos, c-Jun and c-Myc) are involved in regulatory functions involving either DNA replication or control of gene expression. It has been demonstrated that c-*fos* and c-*jun* genes encode nuclear phosphoproteins which are able to complex with each other. The Jun-Fos heterodimeric proteins, called AP-1, recognise specific DNA sequences and mediate transcriptional regulatory activity which has been demonstrated for several genes (for review see Distel and Spiegelman, 1990; Vogt and Bos, 1990; Angel and Karin, 1991). Papillomaviruses with mucosal tropism including HPV16 contain up to three binding sites for AP-1 in their upstream regulatory region (Chong *et al.*, 1990), which have been revealed to be functionally active (Cripe *et al.*, 1990).

The role of c-*myc* in HPV-associated oncogenesis is currently under discussion. Recently, it has been demonstrated that c-Myc binds DNA specifically (Blackwell *et al.*, 1990; Prendergast and Ziff, 1991). However, the set of genes regulated by this nuclear proto-oncogene is unknown, indicating that the direct biochemical actions of the c-Myc protein remain unclear.

Since nuclear proto-oncogenes with potential gene-regulating activity might be involved in human papillomavirus-associated carcinogenesis, we have investigated the *trans*-activating properties of c-Fos, c-Jun and c-Myc on the P97 promoter, which controls the expression of E6 and E7 oncoproteins in HPV16. Using *in situ* hybridisation, we compared these *in vitro* results with proto-oncogene expression patterns in cervical carcinomas.

### Materials and methods

### *Cell culture*

HT-3 cells (kindly provided by P.G. Fuchs, Erlangen, Germany) derived from a cervical carcinoma (Fogh and Trempe,

Correspondence: W Nürnberg, University Clinics Rudolf Virchow, Department of Dermatology, Augustenburger Platz 1, D-13344 Berlin, Germany
Present address: *Institut für Pharmakologie, FU Berlin, Thielallee 69/73, D-14195 Berlin, Germany; †Institut für Medizinische Virologie, University of Zürich, Gloriastrasse 30, 8024 Zürich, Switzerland
Received 8 July 1994; revised 1 December 1994; accepted 5 December 1994

1975) were cultured in RPMI-1640 medium (Gibco Laboratories) containing 10% fetal calf serum (FCS), supplemented with penicillin (100 U $ml^{-1}$) and streptomycin (100 $\mu g\ ml^{-1}$). HT-3 cells are free of endogenous papillomavirus genomes (Yee *et al.*, 1985).

*Construction of plasmids*

The HPV-16 DNA cloned into pBR322 has been described (Dürst *et al.*, 1983) and was kindly provided by H zur Hausen of the Deutsches Krebsforschungszentrum, Heidelberg, Germany. A DNA fragment corresponding to the long control region (LCR) of HPV16 (5'*Eco*RI, 3'*Sau*96I, nucleotides 7453–112) was cleaved from the HPV16 genome and cloned by blunt end ligation into the pCAT-basic plasmid (Promega, Heidelberg, Germany) at the *Xba*I site, generating the pHPV16LCR (Figure 1). Insert-containing clones were identified using restriction analysis.

CAT-reporter plasmids pCMV-CAT, pRSV-CAT and pSVE-CAT containing virus enhancer/promoter sequences are described elsewhere (Artuc *et al.*, 1993). Several expression plasmids for proto-oncogenes and HPV16-derived E2 were used in this study and have been described previously: HPV16-derived E2 expression vector p859 and precursor plasmid p77.01 (Phelps and Howley, 1987), pSVfos (Schönthal *et al.*, 1988) and pSV2-myc-2 (Kingston *et al.*, 1984). The c-*jun* expression vector (p131-1) was constructed introducing the c-*jun* gene derived from RSVc-Jun (Angel *et al.*. 1988) in a pECE expression plasmid (Pharmacia, Freiburg, Germany). The eukaryotic expression plasmid (pECE) was used as a control (Ellis *et al.*, 1986).

For *in situ* hybridisation (ISH), specific probes were generated using a U937 cDNA library. According to standard procedures, polymerase chain reactions were performed to generate specific DNA fragments (Schadendorf *et al.*, 1991). For amplification, primers for c-*fos* (5'-GCCGTCTC-CAGTGCCAACTTCATTCCC-3'; 5'-CTTCACACCGCCA-GCCCTGGAGTAAGC-3') generated a 180 bp DNA fragment, for c-*myc* (5'-AATGTCAAGAGGCGAACACACAA-CGTC-3': 5'-TTTAAGGATAACTACCTTGGGGGCCTT-3') a 135 bp fragment and for c-*jun* (5'-CTCACCTCGCCCGA-CGTGGGGCTGCTC-3'; 5'-TTCGGCCAGGGCGCGCAC-GAAGCCCTC-3') a 180 bp fragment. The identity of the fragments was verified by cloning in a pUC19 vector via the *Sma*I site and dideoxy sequencing. The cellular proto-oncogene DNA (c-*onc*) fragments were cloned in the *Hind*III-and *Eco*RI-digested pSP72 (Promega, Heidelberg, Germany) or pAM18 vectors (Amersham Buchler, Braunschweig, Germany) via sticky end ligation using the *Eco*RI/*Hind*III sites. Plasmids were linearised with *Eco*RI before transcription

**Figure 1** Construction of the pHPV16LCR plasmid. The regulatory segment of the HPV16-LCR was inserted into the *Xba*I site of a CAT plasmid. Binding sites for AP-1 and E2 are indicated. The underlining dashed bar indicates the fragment with strong enhancer activities (Chong *et al.*, 1990).

with T7 RNA polymerase was performed (DIG-RNA labelling kit; Boehringer Mannheim, Germany).

*DNA transfection*

DNA transfection was performed as described previously (Felgner and Ringold, 1989). Briefly, HT-3 cells were seeded into 100 mm plates at a density of 12 000 cells $cm^{-2}$. Cultures were transfected 1 day after they had reached 50–60% confluency. Transfection was performed with Lipofectin (Gibco-BRL, Eggenstein, Germany), with a total of 5 $\mu g$ of DNA per 100 mm dish (2 $\mu g$ of CAT plasmid, 1 $\mu g$ of $\beta$-Gal plasmid and 2 $\mu g$ of expression vector or pUC19) for 15 h. After transfection, cells were incubated for 48 h at 37°C in 5% carbon dioxide atmosphere in 5 ml of RPMI supplemented with 10% FCS. Thereafter, cells were washed with phosphate-buffered saline (PBS) and collected. HT-3 cells were resuspended in 150 $\mu l$ of 0.125 M Tris buffer at pH 7.8 and lysed by freezing and thawing. Supernatants were stored at −20°C. The protein concentration of the lysates was determined according to Lowry *et al.* (1951). After heating the extract at 65°C, the CAT assay was performed.

*CAT assay*

CAT assays were performed as described previously (Gorman *et al.*, 1982). Briefly, cell extracts were incubated with [$^{14}$C]chloramphenicol (40–50 mCi $mmol^{-1}$; NEN, Boston, MA, USA) and 4 mM acetyl coenzyme A (Pharmacia) in 40 mM Tris–HCl, pH 7.8, at 37°C for up to 2 h. Acetylated chloramphenicol derivatives were extracted with ethylacetate and separated by ascending thin-layer chromatography. Thin-layer plates (Schleicher & Schuell, Dassel, Germany) were exposed to the X-ray film for 48 h to localise the acetylated products. Thereafter, the radioactive spots were scraped from the plates for quantification by liquid scintillation. Some *trans*-activation experiments were performed with an alternative standard protocol (Sambrook *et al.*, 1989). After incubation of the cell extracts in the CAT assay buffer containing [$^{14}$C]acetyl coenzyme A (40 mCi $mmol^{-1}$; NEN, Bad Homburg, Germany), 90 $\mu g\ ml^{-1}$ chloramphenicol and 2.5 mM Tris–HCl pH 7.8, quantification was performed in a liquid scintillation fluid using Insta-Fluor (Packard, USA). As determined by independent experiments, both protocols gave the same results (data not shown).

To determine transfection efficiency, $\beta$-galactosidase ($\beta$-Gal) tests were performed. The assay buffer for $\beta$-galactosidase contains 100 nM Hepes, 150 mM sodium chloride, 4.5 mM magnesium hemi-aspartate, 1% bovine serum albumin (BSA), 0.05% Tween 20 pH 7.25 and 3.3 mM chlorophenol-red-$\beta$-D-galactopyranoside (Boehringer Mannheim, Germany). Lysates were diluted 1:25 in substrate buffer and incubated for 60–120 min at 37°C. $\beta$-Galactosidase activities were determined spectrophotometrically at 570 nm and compared with a standard ($\beta$-galactosidase: Promega, Madison, WI, USA).

*In situ hybridisation*

Paraffin-embedded, formalin-fixed sections of primary cervical carcinoma specimens ($n = 9$) were investigated using DNA and RNA *in situ* hybridisation. All tumours were classified as invasive epidermoid (squamous cell) carcinomas of the cervix. HPV16/18 detection was carried out using the commercially available *in situ* Ultradig kit (Boehringer), according to the manufacturer's instructions. In order to investigate the c-*onc* expression in HT-3 cells, the unstimulated cells were incubated in 5% formalin for 20 min. After resuspension in 1% agarose, the cells were paraffin embedded.

For riboprobe *in situ* hybridisation, sections were dewaxed twice in xylol for 20 min. After hydration, the slides were incubated in 0.2 N hydrochloric acid for 20 min. Sections were treated with pronase (0.75 mg $ml^{-1}$ PBS, pH 7.2, for 10 min), followed by fixation in 4% paraformaldehyde for

20 min. Thereafter, slides were incubated in 0.25% acetic anhydride in a 0.1 M triethanolamine (pH 8.0) for 10 min, followed by ethanol dehydration. Prehybridisation was performed using 300 µl of prehybridisation solution [50% formamide; 0.3% sodium chloride, 10 mM Tris-HCl pH 7.5, 10 mM sodium phosphate, pH 6.8, 1 × Denhardt's, 250 µg ml$^{-1}$ yeast t-RNA, 5 mM EDTA, 10 mM dithiothreitol (DTT), 10% dextran sulphate]. Slides were placed in a humidified chamber at 52°C. Three hours later, 100 µl of the antisense or sense solution containing 50 ng of labelled RNA was added to the prehybridisation solution on each section. After 16 h, slides were washed for 4 h in a formamide solution (50% formamide/10 mM DTT/0.5 M sodium chloride, 0.1 M Tris-HCl pH 7.5, 0.1 M sodium phosphate, 0.05 M EDTA pH 8.0, 10 × Denhardt's) at 52°C, followed by two washes with TES (10 mM Tris-HCl pH 7.5, 1 mM EDTA pH 8.0, 0.5 M sodium chloride). After digestion with RNAse A (20 mg ml$^{-1}$) in TES for 10 min at 37°C, sections were washed in TES again at 37°C. Finally, stringent washing steps were performed using 2 × SSC and 0.1 × SSC at room temperature.

Immunohistochemistry was performed according to the protocol of Boehringer Mannheim. Briefly, slides were washed in buffer 1 (100 mM maleic acid, 100 mM sodium chloride, 0.3% Triton X pH 7.5) for 2 min. Sections were preincubated in 1% Blocking Reagent (Boehringer Mannheim, Germany) containing 10% normal sheep serum for 1 h at room temperature. Thereafter, 200 µl of the anti-digoxigenin conjugate (1:500 in buffer 1 containing 10% normal sheep serum) was placed on the slides for 3 h at room temperature. Washing steps were repeated, 5 min in buffer 1 and 5 min in a buffer containing 100 mM Tris-HCl, 100 mM sodium chloride, 50 mM magnesium chloride (pH 9.5), followed by incubation of the sections in colour solution (per ml: 4.5 µl of 4-nitroblue tetrazolium, 4.5 µl of 5-bromo-4-chloro-3-indolyl-phosphate and 3.8 mg of levamisole) at room temperature in the dark. Sixteen hours later, the reaction was stopped by washing the slides in 10 mM Tris-HCl, 1 mM EDTA pH 8.0 for 5 min. Finally, the sections were mounted in Kaiser's glycerin-gelatin without counterstain.

To estimate the amount of specific mRNA expression in the cells and tissues, the signal intensity of the colour was compared after 16 h of immunological detection (−, no signal; +, weak signal; + +, moderate signal; + + +, strong signal).

### Results

### *Activities of various eukaryotic promoters in HT-3 cells*

Sufficient expression of *trans*-activating proteins as well as a sufficient promoter activity of the reporter gene are prerequisites for *trans*-activation experiments. Furthermore, since it has been shown that eukaryotic promoter activities show cell type-dependent activity (Artuc *et al.*, 1993), we determined the activities of various promoters including the human papillomavirus type 16, the cytomegalovirus (CMV), simian virus (SV40) and Rous sarcoma virus (RSV) promoters in HT-3 cells. As shown in Figure 2, all plasmids containing promoter and enhancer sequences were active in HT-3 cells. The CMV promoter displayed the strongest CAT activity (98.5%), followed by RSV (24.1%) and SV40 early promoter (14.2%). The HPV16 (pHPV16LCR) promoter had a basal activity (4.6%) which was one-fifth of that of the RSV promoter. The promoterless plasmid and the SV40 promoter plasmid without enhancer region showed identical low background CAT activities (Table I).

### Trans-*activation of nuclear proto-oncogenes and E2*

In order to investigate the *trans*-activating properties of nuclear proto-oncogenes on the HPV16 P97 enhancer/promoter, we co-transfected the HPV16 reporter plasmid with plasmids expressing the c-Fos, c-Jun, c-Myc and HPV16-E2 proteins in HT-3 cells (Figure 3, Table II). CAT activity was stimulated 3.1- and 3.3-fold by c-Jun and c-Fos respectively. A weak but reproducible stimulation was detectable by co-transfecting the c-*myc* (1.9-fold) and E2 plasmids (2.1-fold). As shown in Figure 3, co-transfection of the control plasmids pECE or p77.01 gave only minor background activity. These data indicate that overexpression of nuclear proto-oncogenes in HT-3 cells resulted in a clear induction of the HPV16 P97 promoter activity.

**Table I** Promoter activates in HT-3 cells. pHPV16LCR, pCMV-CAT, pRSV-CAT, pSVE-CAT, pCAT-promoter and pCAT-basic plasmids were transfected in HT-3 cells

| *Plasmid* | *Relative CAT activity* |
|---|---|
| pCAT-promoter | 2.0 |
| pCAT-basic | 2.1 |
| pSVE-CAT | 14.2 |
| pRSV-CAT | 24.1 |
| pCMV-CAT | 98.5 |
| pHPV16LCR | 4.6 |

Relative CAT activities are expressed as a percentage of acetylation of the supplemented chloramphenicol.

**Figure 2** Promoter activities in HT-3 cells. Transient transfection experiments in HT-3 cells were carried out with pCAT-promoter, pCAT-basic, pSVE-CAT, pRSV-CAT, pCMV-CAT and pHPV16-LCR plasmids. The thin-layer chromatograph shows the results of a representative CAT assay.

**Figure 3** Transcriptional activation of the human papillomavirus promoter/enhancer P97 (pHPV16LCR) by c-*myc* (pSV2-myc-2), c-*jun* (p131-1), c-*fos* (pSVfos) and HPV16-derived E2 (p859). Transient co-transfection experiments were performed in HT-3 cells. A thin-layer chromatograph of a representative CAT assay is shown. Controls: pECE represents the empty control expression vector used for the oncogenes and p7701 the empty expression vector for expression of HPV-E2 (p859).

**Table II** Activation of the human papillomavirus promoter/enhancer P97 (pHPV16LCR) by c-*fos*, c-*jun*, c-*myc* and HPV16-derived E2 (p859) in HT-3 cells

| *Plasmid* | *Number of experiments* | *Relative CAT activity (± s.d.)* |
|---|---|---|
| p77.01 | 3 | 1 |
| p849 (E2) | 3 | 2.1 ± 0.3 |
| c-*fos* | 6 | 3.3 ± 0.5 |
| c-*jun* | 6 | 3.1 ± 0.5 |
| pECE | 6 | 1 |
| c-*myc* | 6 | 1.9 ± 0.5 |

Induction of CAT activity was calculated relative to the basal level obtained with pECE. The transactivating functions of E2 (p859) were quantified relative to the activity of p77.01.

**Table III** Analaysis of HPV16/18 DNA and c-*onc* expression in HT-3 cells and nine invasive cervical carcinomas by *in situ* hybridization

| *Specimens* | c-fos | c-jun | c-myc | *HPV16/18* |
|---|---|---|---|---|
| HT-3 cells | + | ++ | − | − |
| No. 1 | +++ | +++ | − | ++ |
| No. 2 | ++ | ++ | + | ++ |
| No. 3 | ++ | +++ | − | − |
| No. 4 | ++ | ++ | + | ++ |
| No. 5 | +++ | ++ | − | ++ |
| No. 6 | +++ | ++ | + | − |
| No. 7 | +++ | ++ | + | ++ |
| No. 8 | ++ | ++ | − | ++ |
| No. 9 | + | ++ | − | − |

The expression of the riboprobe *in situ* signal was semiquantified by comparing the signal intensity of the colour after 16 h of immunological detection (−, no colour; +, weak signal; ++, moderate signal; +++, strong signal).

### *Proto-oncogene expression in HT-3 cells and HPV-positive cervical carcinomas*

In order to study the c-*onc* and HPV expression in cervical carcinomas, we performed *in situ* hybridisation in HT-3 cells and nine invasive epidermoid (squamous cell) carcinomas of the cervix. The HPV-negative HT-3 cells showed no or only weak expression of c-*myc* and c-*fos* mRNA, while the c-*jun* signal appeared moderate (Table III). Six of nine cervical tissues showed HPV16/18 DNA. In contrast to the normal-appearing cervical mucosa or stroma (Figure 4), c-*fos* and c-*jun* mRNA were expressed in most tumours at high levels. Comparing the signal intensity of c-*fos* and c-*jun*, both proto-oncogenes were expressed in most neoplasms at similar levels. Differences in patterns of proto-oncogene expression between HPV16/18-positive and HPV16/18-negative cervical carcinomas were not detectable. Four of nine tumour specimens expressed detectable amounts of c-*myc* mRNA at low levels.

### Discussion

In recent years, great interest has been focused on transcriptional regulation of human papillomaviruses since this might help to understand the factors involved in the multistep process of carcinogenesis (Cripe *et al.*, 1990; Chong *et al.*, 1990, 1991). In the present study, we have investigated the transcriptional regulation of different nuclear proto-oncogenes on the P97 enhancer/promoter using the cervical carcinoma cell line HT-3. This cervical carcinoma cell line is free of endogenous papillomavirus gene products and shows only a moderate expression of c-*onc* (Table III), making it a good model to study the *trans*-activating properties of HPV and nuclear proto-oncogenes in its natural host cells. After transfection in HT-3 cells, all viral promoters used in our study were transcriptionally active. In agreement with other studies (Chong *et al.*, 1991), the strongest activity was detectable for the CMV enhancer/promoter, which is considered to be ubiquitously active (Boshart *et al.*, 1985). The activity of the HPV16 enhancer/promoter was about one-third that of the pSVE-CAT (4.6% *vs* 14.2%), indicating that the P97 enhancer/promoter is about 5- to 30-fold more active in cervical HT-3 cells than in primary human keratinocytes (Romanczuk *et al.*, 1990). After co-transfection with c-*fos* or c-*jun* expression plasmids, the transcriptional activity in HT-3 cells was stimulated about 3.3- or 3.1-fold respectively. This is in agreement with data described by Cripe *et al.* (1990), who found a 7.9-fold increased transcriptional activation after co-transfection of a P97 reporter plasmid with c-*jun*. In contrast to our study, transfection experiments were performed in undifferentiated mouse F9 teratocarcinoma cells, and the investigators used the P97 reporter plasmid, which contains an LCR core sequence of only 88 nt. It has been reported that shortening of enhancer/promoter regions in reporter plasmids may result in a stronger *trans*-activation (Zobel *et al.*, 1992; Ku *et al.*, 1993), which could be the reason for the stronger *trans*-activation observed by Cripe *et al.* (1990). Infection of keratinocytes with consecutive integration of the HPV genome is normally not associated with fragmentation of the HPV enhancer/promoter region. Therefore, we investigated the *trans*-activating properties with the HPV16 regulative sequence without truncation in accordance with the situation *in vivo*.

c-Fos and c-Jun are the major components of the transcription factor AP-1 (Angel and Karin, 1991). It has been reported that, in contrast to c-Fos, c-Jun proteins are able to form homodimers with DNA-binding properties (for review see Distel and Spiegelman, 1990; Angel and Karin, 1991). However, c-Fos–c-Jun heterodimers have a higher DNA-binding activity than the c-Jun homodimers. In HT-3 cells, the endogenous c-*jun* mRNA level exceeded those of c-*fos* (Table III), suggesting that the induced overexpression of c-Jun might have led to homodimerisation and a consecutive *trans*-activation of the P97 enhancer/promoter.

In agreement with analogous studies which displayed *trans*-activating properties of HPV16-derived E2 on heterologous and homologous reporter plasmids (Cripe *et al.*, 1987; Phelps and Howley, 1987), we observed a weak *trans*-activation of the P97 promoter after p859 co-transfection (Table II), resulting in a viral E2 protein expression.

In contrast to AP-1 and E2, no potential sequence-specific DNA binding sites for the c-Myc protein are detectable on the P97 enhancer/promoter (Figure 1). Since c-Myc showed activating properties on the HPV16 promoter (1.9-fold), we suggest that this effect might be explained by indirect mechanisms which have been demonstrated to be independent from *trans*-activation (Prendergast and Cole, 1989).

To determine the relevance of the observed c-Fos-, c-Jun- and c-Myc-dependent up-regulation of the HPV16 promoter, we performed *in situ* hybridisation studies in nine invasive cervical carcinomas. In contrast to normal-appearing cervical mucosa, all tumours showed a strong expression of c-*fos* and c-*jun*. In agreement with other studies investigating cervical carcinomas by Northern blot technique and histochemically (Bourhis *et al.*, 1990; Cromme *et al.*, 1993), expression of c-*myc* was detectable only in less than 50% of the carcinomas investigated.

Recently, c-*fos* and c-*jun* expression was investigated in normal epidermis (Basset-Seguin *et al.*, 1991). It has been suggested that in those tissues, c-*fos* and c-*jun* transcripts are preferentially located in basal or suprabasal normal keratinocytes and that expression of these proto-oncogenes is linked to differentiation rather than to proliferation (Basset-Seguin *et al.*, 1994; Nürnberg *et al.*, 1994). In malignant cells of squamous cervical epithelium, however, c-*fos* and c-*jun* expression has been found to be distributed in a homogeneous pattern throughout the epithelium (Figure 4), indicating alterations in c-*fos* and c-*jun* gene expression.

In conclusion, our data indicate that overexpression of proto-oncogenes such as c-*fos*, c-*jun* and c-*myc* results in an activation of the P97 promoter *in vitro* and might consecutively, as observed *in vivo*, up-regulate the expression of oncogenic viral proteins. Therefore, deregulation of proto-

**Figure 4** Analysis of HPV16/18 DNA and c-*fos* and c-*jun* mRNA expression in an invasive cervical carcinoma. The sections were hybridised with (**a**) HPV16/18 DNA, (**b**) c-*fos* antisense, (**c**) c-*jun* and (**d**) control *fos* sense RNA probes. The control DNA hybridisation and the sense control for c-*jun* were negative (data not shown). The sections show an HPV-associated tumour infiltrate near HPV-negative mucosal epithelium. This tissue specimen was negative for c-*myc* (no. 1 in Table I). Bar = 20 μm.

oncogene expression might contribute to cervical carcinogenesis.

**Abbreviations:** HPV, human papillomavirus; CAT, chloramphenicol acetyltransferase; c-*onc*, cellular proto-oncogenes; GAL, galactosidase; URR, upstream regulatory region; LCR, long control region; SV40, simian virus; CMV, cytomegalovirus; RSV, Rous sarcoma virus.

**Acknowledgements**
We thank Dr H zur Hausen for cloned HPV 16 DNA and Dr PM Howley for providing the p859 and 77.01 plasmids. The HT-3 cells were kindly provided by Dr PG Fuchs. This study was supported by the Deutsche Forschungsgemeinschaft (Nü 48/1-1).

### References

ANGEL P, HATTORI K, SMEAL T AND KARIN M. (1988). The *jun* proto-oncogene is positively autoregulated by its product, Jun/AP-1. *Cell*, **55**, 875-885.

ANGEL P AND KARIN M. (1991). The role of Jun, Fos and AP-1 complex in cell proliferation and transformation. *Biochim. Biophys. Acta*, **1072**, 129-157.

ARTUC M, NÜRNBERG W, PLATZER M, CZARNETZKI BM AND SCHADENDORF D. (1993). Activity of viral promoters in cultured human skin cells (abstract). *J. Invest. Dermatol.*, **101**, 459.

BASSET-SEGUIN N, ESCOT C, MOLES JP, BLANCHARD JM, KERAI C. & GUILHOU JJ (1991). *C-fos* and *c-jun* proto-oncogene expression is increased in psoriasis: an *in situ* quantitative analysis. *J. Invest. Dermatol.*, **97**, 672-678.

BASSET-SEGUIN N, DEMOLY P, MOLES JP AND 4 OTHERS. (1994). Comparative analysis of cellular and tissular expression of c-*fos* in human keratinocytes: evidence of its role in cell differentiation. *Oncogene*, **9**, 765-771.

BEDELL MA, JONES KH, GROSSMAN SR AND LAIMINS LA. (1989). Identification of human papillomavirus type 18 transforming genes in immortalized and primary cells. *J. Virol.*, **63**, 1247-1255.

BLACKWELL TK, KRETZNER L, BLACKWOOD EM, EISENMANN RN AND WEINTRAUB H. (1990). Sequence-specific DNA binding by the c-Myc protein. *Science*, **250**, 1149-1151.

BOSHART M, WEBER F, JAHN G, DORSCH-HASLER K, FLECKENSTEIN B AND SCHAFFNER W. (1985). A very strong enhancer is located upstream of an immediate early gene of human cytomegalovirus. *Cell*, **41**, 521-530.

BOURHIS J, LE MG, BARROIS M AND OTHERS. (1990). Prognostic value of c-*myc* proto-oncogene overexpression in early invasive carcinoma of the cervix. *J. Clin. Oncol.*, **8**, 1789-1796.

CHONG T, CHAN W-K AND BERNARD H-U. (1990). Transcriptional activation of human papillomavirus 16 by nuclear factor 1, AP1, steroid receptors and a possibly novel transcription factor, PVF: a model for the composition of genital papillomavirus enhancers. *Nucleic Acids Res.*, **18**, 465-470.

CHONG T, APT D, GLOSS B, ISA M AND BERNARD HU. (1991). The enhancer of human papillomavirus type 16: binding sites for the ubiquitous transcription factor oct-1, NFA, TEF-2, NF1, and AP-1 participate in epithelial cell-specific transcription. *J. Virol.*, **65**, 5933-5943.

CHOO K-B, CHONG K-Y, CHOU HF, LIEW L-N AND LIOU, C-C. (1989). Analysis of the structure and expression of the c-*myc* oncogene in cervical tumour and in cervical tumour-derived cell lines. *Biochem. Biophys. Res. Commun.*, **158**, 334-340.

CRIPE TP, HAUGEN TH, TURK JP AND OTHERS. (1987). Transcriptional regulation of the human papillomavirus-16 E6-E7 promoter by a keratinocyte-dependent enhancer, and by viral E2 trans-activator and repressor gene products: implication for cervical carcinogenesis. *EMBO J.*, **6**, 3745-3753.

CRIPE TP, ALDERBORN A, ANDERSON RD AND OTHERS. (1990). Transcriptional activation of the human papillomavirus-16 P97 promoter by an 88-nucleotide enhancer containing distinct cell-dependent and AP-1-responsive modules. *New Biol.*, **2**, 450-463.

CROMME FV, SNIJDERS PJF, VAN DEN BRULE AJC, KENEMANS P, MEIJER CJLM AND WALBOOMERS JMM. (1993). MHC class I expression in HPV 16 positive cervical carcinomas is post-transcriptionally controlled and independent from c-*myc* overexpression. *Oncogene*, **8**, 2969-2975.

CROOK T, ALMOND N, MURRAY A, STANLEY M AND CRAWFORD L. (1989). Constitutive expression of c-*myc* oncogene confers hormone independence and enhanced growth-factor responsiveness on cells transformed by human papilloma virus 16. *Proc. Natl Acad. Sci. USA*, **86**, 5713-5717.

DISTEL RJ AND SPIEGELMAN BM. (1990). Proto-oncogene c-*fos* as a transcription factor. *Adv. Cancer Res.*, **55**, 37-55.

DÜRST M, GISSMANN L, IKENBERG H AND ZUR HAUSEN H. (1983). A papilloma DNA from a cervical carcinoma and its prevalence in cancer biopsy samples from different geographic regions. *Proc. Natl Acad. Sci. USA*, **80**, 3812-3815.

DÜRST M, CROCE CM, GISSMANN L, SCHWARZ E AND HUEBNER K. (1987). Papillomavirus sequences integrate near cellular oncogenes in some cervical carcinomas. *Proc. Natl Acad. Sci. USA*, **84**, 1070-1074.

ELLIS L, CLAUSER E, MORGAN DO, EDERY M, ROTH RA AND RUTTER WJ. (1986). Replacement of insulin receptor tyrosine residues 1162 and 1163 compromises insulin-stimulated kinase activity and uptake of 2-deoxyglucose. *Cell*, **45**, 721-732.

FELGNER PL AND RINGOLD GM. (1989). Cationic liposome-mediated transfection. *Nature*, **337**, 387-388.

FOGH J AND TREMPE G. (1975). In *Human Tumor cells In Vitro*, Fogh J (ed.) pp. 115-159. Plenum Press.

GORMAN CM, MOFFAT LF AND HOWARD BH. (1982). Recombinant genomes which express chloramphenicol acetyltransferase in mammalian cells. *Mol. Cell. Biol.*, **2**, 1044-1051.

HENDY-IBBS P, COX H, EVAN GI & WATSON JV. (1985). Flow cytometric quantification of DNA and c-*myc* oncoprotein in archival bipopsies of uterine cervix neoplasia. *Br. J. Cancer*, **55**, 275-282.

HUGHES R, NEILL WA & NORVAL M. (1989). Papillomavirus and c-*myc* antigen expression in normal and neoplastic cervical epithelium. *J. Clin. Pathol.*, **42**, 46-51.

KINGSTON RE, BALDWIN JR SB AND SHARP PA. (1984). Regulation of the heat shock protein 70 gene expression by c-*myc*. *Nature*, **312**, 280-282.

KU D-H, WEN S-C, ENGELHARD A AND 4 OTHERS. (1993). *C-myb* transactivates cdc2 expression via myb binding sites in the 5'-flanking region of the human cdc2 gene. *J. Biol. Chem.*, **268**, 2255-2259.

LOWRY OH, ROSEBROUGH NJ, FARR AL AND RANDALL AJ. (1951). Protein measurement with folin phenol reagent. *J. Biol. Chem.*, **193**, 265-275.

MATLASHEWSKI G, SCHNEIDER J, BANKS L, JONES, N, MURRAY A AND CRAWFORD L. (1987). Human papillomavirus type 16 DNA cooperates with activated *ras* in transforming primary cells. *EMBO J.* **6**, 1741-1746.

NÜRNBERG W, ROSENBACH TH, SCHADENDORF D AND CZARNETZKI BM. (1994). Changes in proto-oncogene expression during HaCaT keratinocyte differentiation (abstract). *Arch. Dermatol. Res.*, **286**, 172.

OCADIZ R, SAUCEDA R, CRUZ M, GRAEF AM AND GARIGLIO P. (1987). High correlation between molecular alterations of the c-*myc* oncogene and carcinoma of the uterine cervix. *Cancer Res.*, **47**, 4173-4177.

PHELPS WC AND HOWLEY PM. (1987). Transcriptional trans-activation by the human papillomavirus type 16 E2 gene product. *J. Virol.*, **61**, 1630-1638.

PRENDERGAST GC AND COLE M. (1989). Posttranscriptional regulation of cellular gene expression by the c-*myc* oncogene. *Mol Cell. Biol.*, **9**, 124-134.

PRENDERGAST GC AND ZIFF EB. (1991). Methylation-sensitive sequence-specific DNA binding by the c-Myc basic region. *Science*, **251**, 186-189.

RIOU G, BARROIS M, LE MG, GEORGE M, DOUSSAL VL AND HAIE C. (1987). *C-myc* proto-oncogene expression and prognosis in early carcinoma of the uterine cervix. *Lancet*, **i**, 760-763.

ROMANCZUK H, THIERRY F AND HOWLEY PM. (1990). Mutational analysis of cis elements involved in E2 modulation of human papillomavirus type 16 P97 and type 18 P105 promoters. *J. Virol.*, **64**, 2849-2859.

SAMBROOK J, FRITSCH EF AND MANIATIS T. (1989). *Molecular Cloning*. Ford N, Nolan C and Ferguson M (eds) p. 16.64. Cold Spring Harbor Laboratory Press: Cold Spring Harbor, NY.

SCHADENDORF D, TIEDEMANN KH, HAAS N AND CZARNETZKI BM. (1991). Detection of human papillomaviruses in paraffin-embedded condylomata acuminata – comparison of immunohistochemistry, *in situ* hybridization, and polymerase chain reaction. *J. Invest. Dermatol.*, **97**, 549–554.

SCHÖNTHAL A, HERRLICH P, RAHMSDORF HJ AND PONTA H. (1988). Requirement for *fos* gene expression in the transcriptional activation of collagenase by other oncogenes and phorbol esters. *Cell*, **54**, 325–334.

STOREY A, PIM D, MURRAY A, OSBORN K, BANKS L AND CRAWFORD L. (1988). Comparison of the in vitro transforming activities of human papillomavirus types. *EMBO J.*, **7**, 1815–1820.

VOGT PK AND BOS TJ. (1990). Jun: oncogene and transcription factor. *Adv. Cancer Res.*, **55**, 1–35.

YEE CL, KRISHNAN-HEWLETT I, BAKER CC, SCHLEGEL R AND HOWLEY PM. (1985). Presence and expression of human papillomavirus sequences in human cervical carcinoma cell lines. *Am. J. Pathol.*, **119**, 361–366.

YOUNG L, BEVAN I, JOHNSON M AND OTHERS. (1989). The polymerase chain reaction: a new epidemiological tool for investigating cervical human papillomavirus infections. *Br. Med. J.*, **298**, 14–18.

ZOBEL A, KALKBRENNER F, VORBRUEGGEN G AND MOELLING K. (1992). Transactivation of the human c-*myc* gene by c-*myb*. *Biochem. Biophys. Res. Commun.*, **186**, 715–722.

ZUR HAUSEN H. (1986). Intracellular surveillance of persisting viral infections. *Lancet*, **i**, 489–491.

ZUR HAUSEN H AND SCHNEIDER A. (1987). The role of papillomaviruses in human anogenital cancer. In *The Papoviridae*, Vol. 2, Salzman NP and Howley PM (eds) pp. 245–263. Plenum Press: New York.